# *Aedes* (*Ochlerotatus*) *scapularis*, *Aedes japonicus japonicus*, and *Aedes* (*Fredwardsius*) *vittatus* (Diptera: Culicidae): Three Neglected Mosquitoes with Potential Global Health Risks

**DOI:** 10.3390/insects15080600

**Published:** 2024-08-08

**Authors:** Vivian Petersen, Micael Santana, Maria Karina-Costa, Julia Jardim Nachbar, Ines Martin-Martin, Zach N. Adelman, Bianca C. Burini

**Affiliations:** 1 Florida Medical Entomology Laboratory, University of Florida, Vero Beach, FL 32962, USA; vivianpetersen@ufl.edu; 2 Departamento de Parasitologia, Instituto de Ciências Biomédicas, Universidade de São Paulo, Sao Paulo 05508-000, Brazil; micael.santana78@gmail.com (M.S.); markarina7@gmail.com (M.K.-C.); juliajnachbar@gmail.com (J.J.N.); 3 National Center for Microbiology, Instituto de Salud Carlos III, 28029 Madrid, Spain; inesmartin@isciii.es; 4 Department of Entomology and Agrilife Research, Texas A&M University, College Station, TX 77843, USA; zachary.adelman@ag.tamu.edu

**Keywords:** neglected mosquitoes, vector competence, vector capacity, urbanization, climate change, globalization, emerging species

## Abstract

**Simple Summary:**

The main mosquito species capable of transmitting arboviruses belong to the genera *Aedes* spp., *Psorophora* spp., *Anopheles* spp., *Culex* spp., *Mansonia* spp., *Coquillettidia* spp., *Haemagogus* spp., *Sabethes* spp., *Culiseta* spp., and *Wyeomyia* spp. Some neglected mosquito species have the potential to become significant disease vectors due to parameters such as global distribution, rapid adaptation to urban areas, and anthropophilic habits. This review discusses the epidemiological importance and biology of three neglected mosquitoes, *Aedes scapularis*, *Aedes vittatus*, and *Aedes japonicus japonicus*, in the context of vectorial capacity and how urbanization, climate change, and globalization alter disease transmission dynamics and may increase the participation of neglected species in propagating diseases.

**Abstract:**

More than 3550 species of mosquitoes are known worldwide, and only a fraction is involved in the transmission of arboviruses. Mosquitoes in sylvatic and semi-sylvatic habitats may rapidly adapt to urban parks and metropolitan environments, increasing human contact. Many of these mosquitoes have been found naturally infected with arboviruses from the *Alphaviridae*, *Flaviviridae*, and *Bunyaviridae* families, with many being the cause of medically important diseases. However, there is a gap in knowledge about the vector status of newly invasive species and their potential threat to human and domestic animal populations. Due to their rapid distribution, adaptation to urban environments, and anthropophilic habits, some neglected mosquito species may deserve more attention regarding their role as secondary vectors. Taking these factors into account, we focus here on *Aedes* (*Ochlerotatus*) *scapularis* (Rondani), *Aedes japonicus japonicus* (Theobald), and *Aedes* (*Fredwardsius*) *vittatus* (Bigot) as species that have the potential to become important disease vectors. We further discuss the importance of these neglected mosquitoes and how factors such as urbanization, climate change, and globalization profoundly alter the dynamics of disease transmission and may increase the participation of neglected species in propagating diseases.

## 1. Background

Half of the world’s population is at risk of mosquito-borne diseases, which cause more than 700,000 deaths annually [1]. About 3550 known mosquito species worldwide are contained in 2 subfamilies, 11 tribes, and 112 genera [2]. Of these, a few species of mosquitoes encompass species known to be involved in transmitting medically important pathogens, and despite their potential as vectors, are poorly studied. 

Among the principal genera currently involved in disease transmission are *Aedes*, *Culex*, *Culiseta*, *Sabethes*, *Haemagogus*, and *Anopheles* [3]. Two of the world’s most widely distributed and medically important vectors belong to the *Aedes* genus and are the well-known *Aedes aegypti* (Linnaeus) and *Aedes albopictus* (Skuse) [4]. They are present mainly in tropical and subtropical regions, where they are the primary vectors of dengue (DENV), Zika (ZIKV), yellow fever (YFV), and chikungunya virus (CHIKV) [5]. Other aedine mosquitoes have been implicated in arbovirus transmissions, such as *Aedes* (*Ochlerotatus*) *scapularis* (Rondani), *Aedes japonicus* (Theobald), and *Aedes vittatus* (Bigot) [6,7,8,9,10,11,12,13,14,15]. In the genus *Culex*, the mosquitoes of the *pipiens* complex, *Culex pipiens* (Linnaeus), *Cx. quinquefasciatus* (Say), and *Culex tarsalis* are also among the most prominent vectors, transmitting a range of pathogens, including West Nile virus (WNV), avian malaria parasites, and the filarial worm *Wuchereria bancrofti* [16,17,18]. Among the *Anopheles* mosquitoes, about 60 known species within this genus are vectors of malaria parasites. Among important anopheline vectors, *Anopheles gambiae* (Giles) is considered one of the most epidemiologically important species in sub-Saharan Africa, along with *Anopheles arabiensis*, *Anopheles coluzii*, and *Anopheles stephensi* (Liston), the latter being a primary vector in urban India, initially confined to countries in South Asia and parts of the Arabian Peninsula, and now having recently invaded Africa, causing significant concern [18,19,20]. 

The genera *Haemagogus* and *Sabethes* are known to encompass the main vectors of wild yellow fever in Brazil, with the main vector species *Haemagogus janthinomys*, *Haemagogus albomaculatus*, *Haemagogus leucocelaenus*, *Sabethes chloropterus*, *Sabethes soperi*, and *Sabethes cyaneus* [21,22]. In addition to those mentioned above, other species belonging to other genera may be involved in the cycles of different viruses and deserve attention regarding their potential as a vector, such as the mosquitoes of the genus *Psorophora* that were found naturally infected with the Rocio virus in Brazil and with Venezuelan Equine Encephalitis (VEEV) in North America, both during an epidemic [23,24]. Recently, the mosquito *Wyeomyia bourrouli* (Lutz) was found for the first time infected with CHIKV in Brazil at the epicenter of an epidemic outbreak, along with other species, including *Ae. aegypti* and *Ae. albopictus* [25]. Regarding the genus *Mansonia*, three species, *Mansonia uniformis*, *Mansonia bonneae*, and *Mansonia dives*, were incriminated as the vectors of the worm *Brugia malayi* in Southern Thailand, a pathogen that can cause fever, inflammation of the lymph nodes, and chronic lymphedema [26]. In addition, *Mansonia humeralis* was recently found naturally infected with dengue and Mayaro viruses in Rondônia, Brazil [27]. In the genus *Culiseta*, the species *Culiseta melanura* is the primary enzootic vector of the eastern equine encephalomyelitis virus (EEEV) throughout eastern North America [28]. Lastly, in the genus *Coquillettidia*, mosquitoes tested positive for *Plasmodium* spp., which causes avian malaria in Cameroon, West Africa [29]. *Coquillettidia perturbans* was implicated as a vector species of eastern equine encephalitis as it was found naturally infected with this virus, and it has feeding patterns that favor transmission between hosts [30,31].

**Figure 1:**
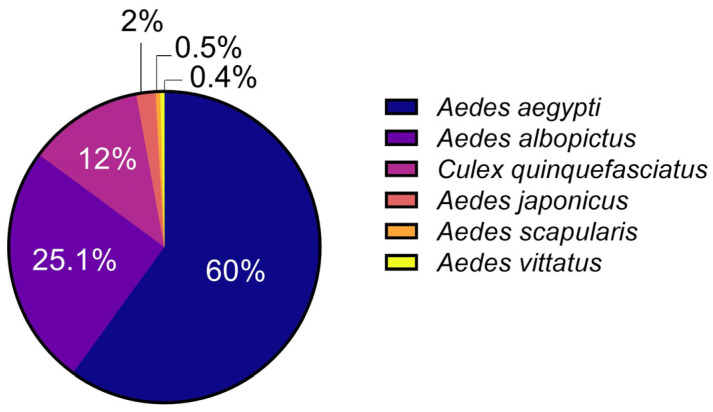
Proportion of the number of scientific journals present on the Pubmed server in the last ten years regarding the species *Aedes aegypti*, *Aedes albopictus*, *Culex quinquefasciatus*, *Aedes japonicus*, *Aedes vittatus*, and *Aedes scapularis*.

Given the vast gap in knowledge of emerging vector mosquitoes, this review discusses the current state of knowledge regarding emerging mosquito species implicated in disease transmission and the factors that may influence their vectorial capacity. We focused on *Ae. scapularis*, *Ae. j. japonicus*, and *Ae. vittatus* as, based on the literature available, these species could participate in epidemics or have the potential to start one due to specific characteristics that will be presented. However, their current knowledge is far from what is available for primary vector species such as *Ae. aegypti*, *Ae. albopictus*, or *Cx. quinquefasciatus*, and for that, we refer to them as neglected mosquitoes. A search for papers published in the last ten years was conducted on the scientific periodic server Pubmed [32] on the date 8 March 2024 using the keywords: *Aedes aegypti*, *Aedes albopictus*, *Culex quinquefasciatus*, *Aedes scapularis*, *Aedes japonicus*, and *Aedes vittatus*. This search rendered a significant difference in the number of publications between these species (Figure 1). The *Ae. aegypti* mosquito presented the highest percentage of published papers, with 60%. In comparison, the species *Ae. albopictus* accounted for approximately 25.1%, *Cx. quinquefasciatus* for 12%, while the species *Ae. j. japonicus*, *Ae. vittatus*, and *Ae. scapularis* accounted for the lowest numbers, at 2%, 0.5%, and 0.4% of total papers published, respectively (Figure 1). These data illustrate the significant disparity in the number of papers available for the main vectors compared to *Ae. j. japonicus*, *Ae. vittatus*, and *Ae. scapularis*, supporting the neglect in the study of those species. We aim to bring awareness about their role in disease transmission and their potential to become critical epidemiological vectors with the world and climate change. 

## 2. Vector Capacity and Vector Competence

**Figure 2:**
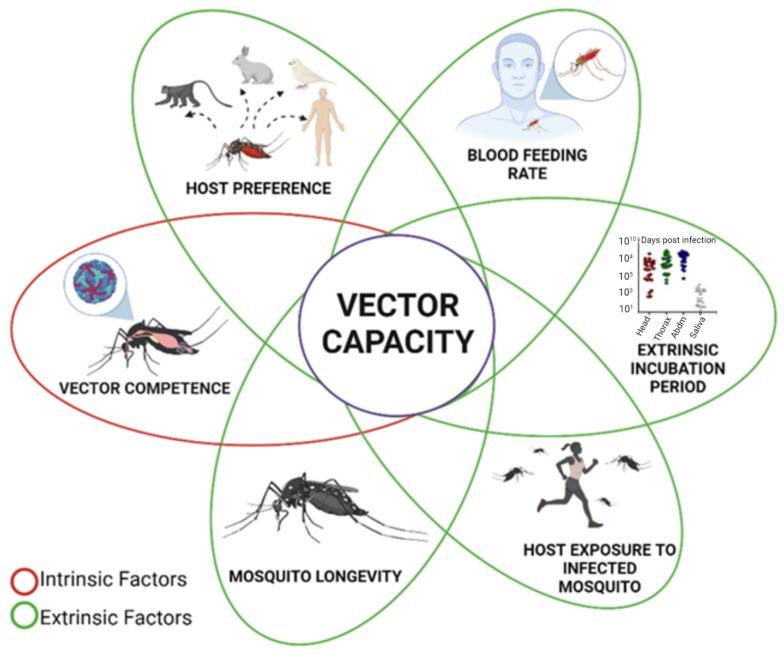
Factors related to mosquito vectorial capacity. Many factors contribute to determining mosquito vectorial capacity. Intrinsic factors, like vector competence, remain constant over time (highlighted in red), although certain environmental conditions may affect them. In contrast, extrinsic factors (highlighted in green) can be modified by location and time, including host preference, blood feeding rate, the extrinsic incubation period, host exposure to infected mosquitoes (mosquito density), and mosquito longevity.

The relationship between pathogens and hosts is complex and dependent on intrinsic and extrinsic factors that determine overall vectorial capacity (Figure 2), defined as the number of new infections that result per starting infection [33]. Extrinsic factors are related to environmental factors, density and the abundance of the mosquito and its host, and the frequency of mosquito–host contact. Intrinsic factors are related to immunity, virus replication in mosquito tissues, longevity, hematophagy preferences for their host, and the timing of blood feeding whenever these factors have a genetic basis (Figure 2) [34]. 

Vectorial capacity is a broader term that includes vector competence. Not all vectors showing competence for certain viruses in laboratory experiments will demonstrate sufficient vector capacity to sustain transmission. Thus, for a mosquito species to have enough vectorial capacity to sustain epidemic or endemic transmission, the pathogen must replicate in its body and be transmitted through saliva during a blood meal. This competent mosquito must have a host preference for the vertebrate carrying the etiological agent, and both the host and the mosquitoes must be in sufficient numbers for contact to guarantee transmission in both directions. Finally, that mosquito must live long enough for the pathogen to complete its life cycle and be infectious to the next host. Another factor influencing the mosquito’s susceptibility to transmitting arboviruses is its intestinal microbiota composition [35]. The composition of the gut microbiota of mosquitoes can also affect their susceptibility to transmitting pathogens [36]. Some intestinal bacteria are associated with the production of antiviral proteins, and others may act to activate the mosquito’s innate immune system [37]. This topic is outside the scope of this work, and excellent reviews can be found elsewhere [36,38,39].

Many species of mosquitoes besides *Ae. aegypti*, *Ae. albopictus*, and *Cx. quinquefasciatus* are often ignored in research and control efforts. However, they are fundamental in the transmission of diseases in certain regions. Comprehensively understanding all factors determining vectorial capacity contributes to predicting which species have the potential for arbovirus transmission, enabling targeted vector control measures to inhibit the proliferation of vectors that could lead to new epidemics.

## 3. Potential Emerging Vectors

Despite the scarcity of information on neglected species, three mosquito species, *Aedes* (*Ochlerotatus*) *scapularis* (Rondani), *Aedes japonicus japonicus* (Theobald), and *Aedes* (*Fredwardsius*) *vittatus* (Bigot), deserve attention for their potential as vectors. The choice of these three species was based on known parameters related to vectorial capacity, such as adaptation to urban environments, anthropophilic behaviors, vector competence for medically important viruses, and the expansion of distribution. Other mosquito species exhibit one or more of these parameters that are important in vector capacity; however, they lack others. For example, *Aedes koreicus* has anthropophilic [40] habits, but its distribution is restricted to some European and Asian countries [41]; moreover, this species has not been found naturally infected with medically important arboviruses. Another example is *Aedes vigilax*, which also exhibits anthropophilic habits and a preference for other mammal species [42], but it also has a limited distribution to some Asian countries and Oceania [43]. 

### 3.1. Aedes (Ochlerotatus) scapularis 

#### 3.1.1. Distribution

**Figure 3:**
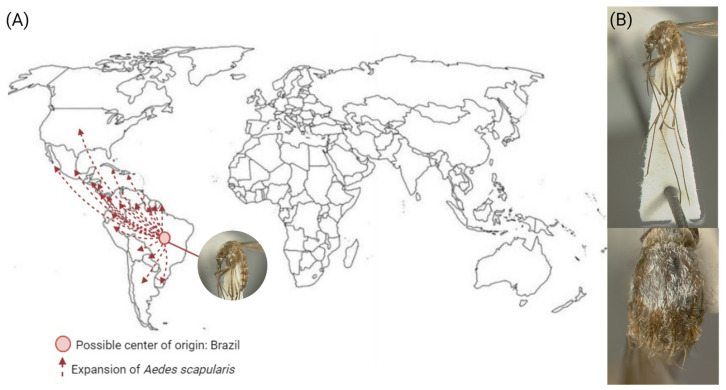
*Aedes scapularis* expansion and morphological characteristics. (**A**) The red dot represents the possible center of origin (Brazil), and the dotted red arrows show the countries where *Ae. scapularis* has been found. (**B**) Top: lateral full-body view. Botton: thorax depicting a large patch of silver scales. Specimens were collected near Belem, Para, Brazil, and adapted from [43]. Images of the *Ae. scapularis* were provided by the Walter Reed Biosystems Unit (WRBU) and the Smithsonian Institution and reproduced with permission from Dr. Yvonne Linton.

*Aedes scapularis* is a neotropical species widely distributed in Central America and South America [6]. The mosquito *Ae. scapularis* likely has South America as its center of origin, being initially collected in Belém do Pará, Brazil. Over the years, this species has expanded its distribution to North America (Figure 3) [43,44]. The map in Figure 3 was created based on information made available by the Walter Reed Biosystematics Unit (WRBU) about the type of localities and the locations where *Ae. scapularis* is currently occupying. The WRBU maintains the most extensive online insect vector surveillance database, with approximately 0.95 million entries, and the data used by the WRBU are results from museum collections, the literature, and global biosurveillance, covering mosquitoes, ticks, sand flies, and mites and providing a reliable source for the presence of different species around the world. 

Collections of larval and adult stages of *Ae. scapularis* in Miami-Dade and Broward Counties performed in 2016 through 2019 in Florida, USA, showed highly similar COI (mitochondrial gene Cytochrome C Oxidase Subunit I) sequences and, along with its absence in that locality since 1945, suggest a new introduction and establishment from a source population in the Neotropics (Figure 3A) [44]. 

#### 3.1.2. Morphological Identification

Both male and female adult *Ae. scapularis* can be distinguished from *Ae. aegypti* by their scutal ornamentation, having a large patch of silver scales (Figure 3B), while *Ae. aegypti* has silver scales in a lyre shape. Differences in the size of the silver scales on the scutum and the gonocoxite claspet have been observed in populations of this mosquito [2,45]. It is also quickly differentiated from the *Ae. albopictus* mosquito, which has a line of pale scales on its posterior scutum [46]. Regarding its taxonomic status, it has been investigated whether this species belongs to a species complex, as it presents great genetic and morphological variability. A high proportion of haplotypes of the mitochondrial COI gene were found in populations collected in Brazil with low sequence similarity, suggesting that this species is genetically polymorphic. Despite high genetic polymorphism and geographic population structuring, to date, *Ae. scapularis* has not been found to represent a complex of closely related species [44,47,48]. Polymorphism can also be observed in the morphology of this mosquito, as differences in the size of the silver scales on the scutum and the gonocoxite claspet have been observed in populations of this mosquito [2,6,45]. The high plasticity in disease transmission exhibited by polymorphic species and their significant potential to initiate the transmission of new pathogens underscores the importance of closely monitoring *Ae. scapularis* to become a potentially important vector. 

#### 3.1.3. Biology

*Aedes scapularis* was previously considered rural and sylvan; however, it has adapted to urban and human-modified landscapes, displaying synanthropic characteristics [49,50]. This mosquito is increasingly urbanizing, being present in parks and domestic environments. Females of this species were analyzed during an arbovirus epidemic in Brazil, showing an anthropophilic feeding preference since it was revealed that 83% of the mosquitoes were engorged with blood from humans [51]. In addition, this species was also found in different settings, both rural and urban regions, with a wide distribution that demonstrated the capacity to adapt to various environments [40].

*Aedes scapularis* lives for approximately 26 days under laboratory conditions and usually requires more than one blood meal to complete the gonotrophic cycle [52], an important factor that increases host contact. The need for multiple blood feedings and plasticity in adapting to different environments, together with their anthropophilic behavior, indicates that *Ae. scapularis* has biological characteristics that are important for disease transmission. 

#### 3.1.4. Vector Competence and Capacity 

*Aedes scapularis* has been found naturally infected with several arboviruses, including YFV, Melao, Ilheus, Venezuelan equine encephalitis, St. Louis encephalitis viruses, and *Dirofilaria immitis* [6,7,53]. The incrimination, as a critical component of the transmission cycle, happened for YFV, where *Ae. scapularis* was considered a secondary vector in the sylvatic cycle of YFV during the epidemic that began in 2017 in southeastern São Paulo, Brazil, among non-human primates. Factors contributing to this conclusion were a YFV-positive pool of *Ae. scapularis*, the observation that this species was found to be the most abundant in the region, a blood feeding preference for human and non-human primates, and the presence of several YFV-positive non-human primates—mainly of the genus *Callithrix* spp.—in the served region [54]. *Aedes scapularis* was also previously found naturally infected with YFV in Brazil during an epidemic that occurred 17 years prior to the non-human primate epidemic [53].

While these studies are beneficial, they represent a single example. Populations of *Ae. scapularis* from different regions should be similarly assessed for a comprehensive understanding of potential vector status. To our knowledge, there are no records on the competence of this mosquito species to any medically important viruses such as DENV, ZIKV, and CHIKV (Table 1), complicating the prediction of whether this species can participate in or sustain disease transmission. Comparison studies of vector competence with those viruses using populations from different places could help elucidate intrinsic differences between populations and prevent outbreaks if that species is competent for one or more viruses. 

### 3.2. Aedes japonicus japonicus

#### 3.2.1. Distribution

**Figure 4:**
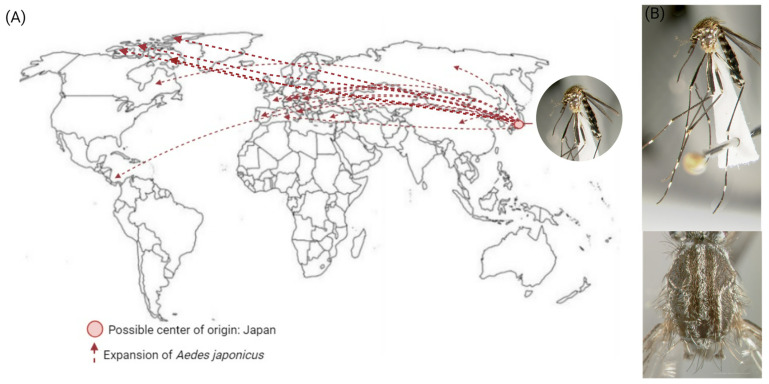
*Aedes japonicus japonicus* expansion and morphological characteristics. (**A**) The red dot represents the possible center of origin (Japan), and the dotted red arrows show the countries where *Ae. j. japonicus* has been found. (**B**) Top: lateral full-body view. Botton: thorax depicting the bronze-colored lyre-shaped scales. Specimen collected in Tokyo, Honshu, Japan. Deposited at the Natural History Museum, London, England, United Kingdom (NHMUK); pictures were adapted from [74]. Images of the *Ae. j. japonicus* were provided by the Walter Reed Biosystems Unit (WRBU) and the Smithsonian Institution and reproduced with permission from Dr. Yvonne Linton.

*Aedes japonicus japonicus* (Theobald, 1901), known as the rock pool mosquito or the Asian bush mosquito, is a species common in Japan, Korea, and China; however, it is increasingly expanding around the world, invading countries in Europe and the Americas, including Canada and the US [59,62,63,64,65,66,67,68]. In Europe, this mosquito was first detected in the 2000s in France, and since then, it has been found in West Germany, Luxembourg, French Alsace, and southwards to Switzerland, Liechtenstein, Austria, Spain, and Italy. In the US, it was first detected in Connecticut in 1998 and rapidly expanded throughout North America within 15 years to places such as Illinois, New York, Connecticut, and New Jersey [69] and reaching parts of Canada (Figure 4A) [70]. In addition to these areas, mathematical models using maximum entropy modeling to estimate the potential distributions of *Ae. j. japonicus* predict that this mosquito could expand even more and colonize and develop in Alaska and Latin America [71]. Using climate change-based mathematical simulation models, *Ae. j. japonicus* population density is predicted to rise and spread [72]. This mosquito is more adapted to temperate regions, suggesting that it would not manage to survive in warmer areas, but mathematical models based exclusively on physiological data indicate a further spread of this species beyond temperate limits [72]. This tolerance of temperate climates supported the expansion of this species to a multitude of habitats with a successful establishment outside its origins [73].

#### 3.2.2. Morphological Identification 

*Aedes japonicus japonicus* adults have lyre-shaped scales on the scutum but are bronze-colored instead of silver like *Ae. aegypti* (Figure 4B), and the larvae have a spiculated anal saddle and a linear arrangement of branched frontal setae, a feature that distinguishes this species from other mosquitoes in North America [64,75]. Four allopatric subspecies compose the *Ae. j. japonicus* complex, and although morphologic diversity is not enough to identify the adults of the subspecies, genetic analyses using two mitochondrial loci and a nuclear locus revealed that they are substantially distinct, and a taxonomic revision could raise some of the subspecies to species [76]. 

#### 3.2.3. Biology

*Aedes japonicus japonicus* females display crepuscular habits regarding their blood feeding. A study conducted in the USA indicated that this species shows a preference for mammals, including humans, over other animals [77,78]. *Ae. j. japonicus* naturally utilizes holes in rocks as breeding sites. Additionally, larvae can be found in rainwater puddles, holes in tree trunks, and various man-made containers such as gutters and other receptacles found in urban environments, where this species is highly adaptable. Larval forms of this species have also been discovered in tire storage sites in France and in tire trading companies in Belgium [66,73], demonstrating high adaptability in domestic settings. *Ae. j. japonicus* thrives in temperate climates and can survive adverse conditions, including regions with cold and harsh winters, where it can also develop in high-altitude areas [79,80].

#### 3.2.4. Vector Competence and Capacity

Besides WNV, *Ae. j. japonicus* has been found naturally infected with La Crosse encephalitis virus, the cause of the most reported pediatric arboviral encephalitis in the US, and Cache Valley virus, also a cause of encephalitis associated with a single human fatal case in the US [81,82]. However, the fact that medical laboratories rarely test for them can lead to underestimating the medical importance and the true incidence, highlighting the need to further study their capacity for those viruses [8,11].

*Aedes j. japonicus* has vector competence for a broad range of arboviruses such as JEV, SLEV, EEEV, LACV, ZIKV, CHIKV, DENV, and WNV (Table 1) [10,14,55,59,83,84,85,86] although different levels of competence were found among them. This versatility in vector competence has only been previously shown for *Ae. aegypti* and *Ae. albopictus*. Taken together, these data suggest that *Ae. j. japonicus*, with its high adaptability to different environments coupled with recent expansion beyond the native range and competence for a broad range of medically important viruses, poses a significant risk to public health. Comprehensive studies are needed to elucidate the specific host preference and vector competence of *Ae. j. japonicus* in different localities to different pathogens aiming to address region-specific behavior from this mosquito. This knowledge is crucial for devising targeted strategies to prevent future WNV and other arbovirus outbreaks. 

### 3.3. Aedes (Fredwardsius) vittatus

#### 3.3.1. Distribution 

**Figure 5:**
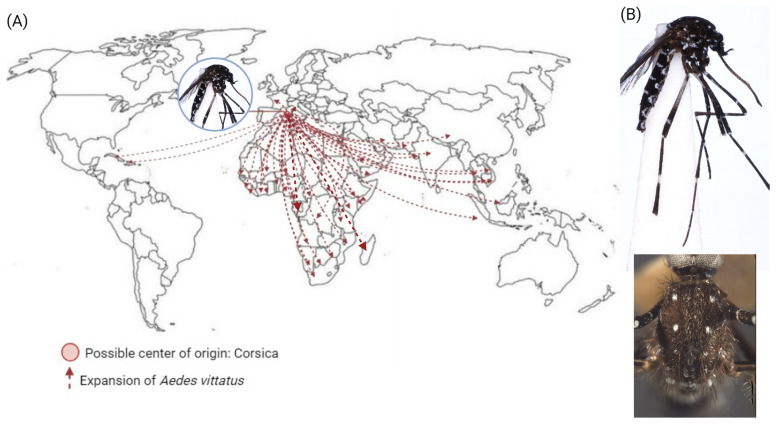
*Aedes vittatus* expansion and morphological characteristics. (**A**) The red dot represents the possible center of origin (Corsica, France), and the dotted red arrows show the countries where *Ae. vittatus* has been found. (**B**) Top: lateral full-body view. Botton: thorax depicts three pairs of round, silvery white spots. Specimens were collected in Cuba in a CO_2_-baited CDC light trap at Naval Station Guantanamo Bay on 18 June 2019, and pictures were adapted from [87,89]. Images of the *Ae. vittatus* were provided by the Walter Reed Biosystems Unit (WRBU) and the Smithsonian Institution and reproduced with permission from Dr. Yvonne Linton.

*Aedes* (*Fredwardsius*) *vittatus* was initially found in Corsica, an island in the Mediterranean Sea, located southeast of France and west of Italy, and later in all five regions of Africa—including Senegal, Sudan, Ethiopia, and Kenya—and countries in Asia, such as China, Bangladesh, Iran, Nepal, India, Vietnam, Malaysia, Saudi Arabia, Sri Lanka, and Thailand. In Europe, it is restricted to the western Mediterranean region, being found in France, Italy, Portugal, and Spain. In 2019, during routine entomological inspections, *Ae. vittatus* was detected in the Americas in the Dominican Republic and Cuba [15,87,88]. Figure 5A demonstrates the broad range of distribution of *Ae. vittatus* reaching several countries in tropical and some subtropical regions of the world. 

#### 3.3.2. Morphological Identification

This mosquito has a dark beak with pale yellowish scales, its scutum has three pairs of small white spots, and its scutellum has white scales on all three lobes; this characteristic sets it apart from other aedine mosquitoes. Regarding its abdomen, tergum I has a large median white spot, and its legs have all dark tibias (Ti-I-III) with a sub-basal white spot [87]. The distinctive feature of *Ae. vittatus* lies in the arrangement of three pairs of small, round, silvery-white spots on its scutum, setting it apart from other aedines (Figure 5B).

#### 3.3.3. Biology

Historically, this species is mainly considered a rock-hole breeder; however, breeding habitats can differ depending on the locality, whereas in some parts of Nigeria, India, and Pakistan, the breeding of this mosquito has been predominantly found in artificial containers such as tires, bottles, cups, and potted plants in peridomestic habitats [90,91], demonstrating that this species is urbanizing. Studies have attested that *Ae. vittatus* is an aggressive human biter and shows a strong preference for human blood over other animals such as cattle, pigs, and chickens [91]. In relation to the gonotrophic cycle, this species has a blood feeding interval of 4 to 5 days [92].

#### 3.3.4. Vectorial Competence and Capacity

YFV and ZIKV were isolated from *Ae. vittatus* in Senegal (Table 1). In collections performed in forests, savannahs, agricultural areas, villages (indoor and outdoor), and barren land cover sites, *Ae. vittatus* was found naturally infected with ZIKV and was implicated in transmission in a domestic environment in southeast Senegal [58]. In the same country, YFV was isolated from *Ae. vittatus* from monospecific pools, although not as frequently infected as *Aedes furcifer* (Edwards), a critical YFV vector in the region [93]. These data demonstrate a possible implication of this mosquito on the cycle of both ZIKV and YFV in Senegal. Entomological surveys and mosquito viral infection testing during outbreaks in different regions could help elucidate the involvement of this species in transmission cycles. 

Although only ZIKV and YFV were isolated from wild mosquitoes, *Ae. vittatus* competence has been tested in laboratories for several arboviruses (Table 1) [12,15,94,95]. Regarding ZIKV, this mosquito displayed high infection and dissemination rates and tested positive for this virus in the saliva [94]. Kenya’s population of *Ae. vittatus* was tested for the East/Central/South Africa (ECSA) CHIKV strain, showing high susceptibility and moderate dissemination and transmission. Accordingly, *Ae. vittatus* from Senegal, on the opposite side of Africa, demonstrated higher susceptibility, dissemination, and transmission rates than *Ae. aegypti* to the previously tested ECSA and West Africa strains [13]. Taken together, these data demonstrate that *Ae. vittatus* is a competent vector for CHIKV. In addition, *Ae. vittatus* competence was tested for other arboviruses such as Japanese Encephalitis, West Nile, Chandipura, and Chittoor viruses. Although viral replication was demonstrated of all these viruses through intrathoracic injection, vector competence in oral feeding experiments was only demonstrated for WNV [15]. The transovarian transmission of DENV is an important phenomenon that maintains the occurrence of this virus during interepidemic periods of this disease. This phenomenon was observed to occur in *Ae. vittatus* in the Rajasthan district of India, in which a vertical transmission rate of 20% was recorded in mosquitoes analyzed using the Indirect Fluorescence Antibody Test [96]. 

The wide distribution, vector competence for several arboviruses, adaptation to urban environments, and involvement in outbreaks of YFV and ZIKV make *Ae. vittatus* a vector that should be closely monitored using surveillance methods to prevent new transmission cycles.

#### 3.3.5. Other Neglected Mosquitoes

Other mosquito species have been found naturally infected or had their vector competence tested in a laboratory with medically important pathogens, such as *Aedes furcifer*, *Aedes taylori* (Edwards), and *Aedes luteocephalus* (Newstead), which were found naturally infected with DENV-2 in southwestern Senegal [95,97] or *Aedes koreicus*, which was shown to be competent to CHIKV in laboratory infections [98], to name a few. Those studies highlight the potential of those species to participate in or establish disease transmission wherever they are found. However, it is impossible to establish their role in epidemic episodes solely based on those studies. For those mosquitoes and many other neglected species, no comprehensive analysis can appropriately evaluate all parameters involved in calculating the vectorial capacity, thus making it impossible to understand their role in disease transmission by themselves or along with primary vectors.

## 4. Factors That Favor the Emergence of Vectors: Climate Change, Globalization, Urbanization

There is an increase in the incidence of arbovirus transmission in places with no records before [99]. Some factors can answer why transmission is increasing: changes in global temperature, urbanization, the globalization of trade, international travel, increased dispersion, and the distribution of invasive mosquito species [12,99,100]. To contain the advance of these diseases, understanding the dynamics of pathogen transmission and the factors contributing to the spread of diseases is of utmost importance. 

Climate change, primarily when related to increasing temperatures, can alter the dynamics of the transmission of pathogens by mosquitoes, as these vectors can begin to develop in places where it was not favorable before due to low temperatures [100]. The increase in global temperatures decreases the extrinsic incubation time of the virus, speeds up the development of immature stages of mosquitoes, and summers with more rainfall lead to more breeding sites [101,102]. Climate change makes it easier for invasive species to adapt to other territories [103]; however, human actions also contribute to mosquito colonization in new places, as many species were introduced through containers containing diapause eggs [104]. It has already been observed that immature forms of *Ae. albopictus* in artificial containers have spread considerably along US highways through human activity [105]. The same phenomenon was observed for the species *Ae. j. japonicus* in the US, as it was observed that the expansion of this species of mosquito is strongly associated with areas inhabited by humans, as there is observation between the genetic distance (microsatellite loci) between Virginia mosquitoes and the distance along roads, even after considering geographic distance [106].

Globalization and climate change can increase the number of arbovirus cases [107] and the distribution and adaptation of invasive species [108], increasing the likelihood that secondary species of mosquitoes will play an essential role in disease transmission. For these reasons, it is crucial to maintain effective entomological and viral surveillance campaigns to facilitate control efforts where they are most needed. Furthermore, it is not yet known whether emerging vectors are being introduced to other areas or whether the native mosquitoes come into contact with newly introduced viruses, can establish a pathogen–host cycle, and become important in the transmission of these etiological agents of diseases. The increase in the flow of people for tourism, work, and migration also modifies the dynamics of arbovirus transmission. With the rise in international travel, the risk of introducing pathogens into regions where they did not occur before or have become extinct increases [109]. This is the case of cities in Florida, US, that registered imported and locally acquired dengue cases in 2020, probably from tourists, immigrants, or traders from other countries [110]. With the introduction of new pathogens, there is an increased possibility of species other than *Ae. aegypti* and *Ae. albopictus* to transmit emerging viruses. 

Urbanization consists of advancing cities into new areas, where the environment is modified to accommodate local population growth and arise from rural regions [111]. Urbanization can lead to the domestication of mosquito species that can adapt to artificial environmental changes, as the urban environment serves as a refuge and facilitates the proliferation of these vectors [112] since there are fewer predators available, the formation of new breeding sites, and the availability of a human host for blood feeding [113]. In addition, the invasion of new areas can expose viruses that previously only circulated in a particular region, thus modifying their transmission dynamics. In cities, there is a phenomenon called heat islands, in which urban areas experience higher temperatures than in rural, sylvan environments, which can lead to the faster development of the life cycle of vectors [114]. *Ae. aegypti* infected with DENV and exposed to temperatures similar to those found in heat islands had blood feeding, oviposition, and virus development positively associated with temperature [114]. For every one °C increase in the average monthly temperature in Taiwan, the risk of dengue transmission is predicted to increase by 1.95 in the population of that country, according to predictions based on statistical models that consider climate change [115].

Finally, the invasion of different mosquito species in urban parks can be a problem for public health, as they can potentially be vectors of several arboviruses and remain neglected in their control, enhancing disease transmission [116]. Finally, the shift from zoophilia to anthropophilia enables the introduction of new pathogens to humans that can become of medical concern [117].

## 5. Conclusions

It is currently known that different species of mosquitoes, in addition to the primary vectors, may have significant vectorial capacity for arboviruses and other etiological agents of diseases and participate in disease transmission. For a species to be considered a vector of pathogens for humans, parameters such as vector competency, anthropophilic behavior, human contact, and population density must be taken into account and can be quantitatively measured. Secondary and emerging vector species are relatively neglected, and most of this ecological/behavioral information remains unknown and should be monitored in vector biology campaigns considering these parameters. *Ae. scapularis*, *Ae. j. japonicus*, and *Ae. vittatus* are three neglected species that, based on some crucial vectorial capacity parameters, represent a potential threat to becoming important disease vectors. A complete understanding of the host-pathogen interactions and ecology of these species is needed for targeted control and disease outbreak prevention. There are outstanding questions that still need to be addressed. Since these three species are invading new places, where are they likely to go next? Where are they most likely to become a public health problem for the places they have invaded? Can they amplify existing public health issues caused by well-recognized *Ae. aegypti* and *Ae. albopictus*, thereby exacerbating current problems? To what extent do neglected species have the potential to compete with or coexist alongside *Ae. aegypti* or *Ae. albopictus*? Overlooking the less studied species could inadvertently undermine vector control strategies and compromise efforts to mitigate the impact of mosquito-borne diseases.

Many other mosquito species have left us wondering and questioning their vector status; however, a more extreme lack of knowledge makes it hard to judge and highlight the need for more studies. Some factors can amplify the danger of secondary vector species transmitting arboviruses; globalization, climate change, and urbanization enable the establishment of both viruses and mosquito species in different regions of the world, change disease transmission dynamics, and present new challenges in vector-borne disease control. These new challenges promote the need to intensify entomological surveillance, studies of vector competence, and the promotion of vector control programs to prevent the spread of vector mosquitoes.

## Figures and Tables

**Table 1:** Vector competence and viruses found in *Aedes japonicus*, *Aedes scapularis*, and *Aedes vittatus* species.

Virus	*Aedes scapularis*		*Aedes japonicus*		*Aedes vittatus*		
	Vector Competence	Naturally Infected	Vector Competence	Naturally Infected	Vector Competence	Naturally Infected	References
Chikungunya	-	-	✓	-	✓	-	[13,14]
Dengue	-	-	✓	-	-	-	[14]
Japanese Encephalitis	-	-	✓	-	-	-	[55]
Rift Valley Fever	-	-	✓	-	-	-	[56]
St. Louis Encephalitis	-	✓	✓	-	-	-	[10,57]
Zika	-	-	✓	-	-	✓	[58,59]
Yellow Fever	-	✓	-	-	-	✓	[53,54,60]
Venezuelan Equine Encephalitis	-	✓	-	-	-	-	[61]
West Nile	-	-	-	✓	✓	-	[9,15]

## Data Availability

The original data presented in the study (Figure 1) are openly available at https://pubmed.ncbi.nlm.nih.gov/ (accessed on 5 June 2024). Restrictions apply to the availability of mosquito pictures (from Figure 3, Figure 4 and Figure 5). Pictures were obtained from the Walter Reed Biosystematic Unit (WRBU) and the Smithsonian Institution and are available (at https://wrbu.si.edu/, accessed on 6 March 2024) with the permission of Dr. Yvonne Linton.

## Abbreviations

DENV	Dengue virus
EEEV	Eastern Equine Encephalitis Virus
CHIKV	Chikungunya virus
JEV	Japanese Encephalitis
LACV	La Crosse Encephalitis Virus
MAYV	Mayaro virus
OROV	Oropoche virus
RVFV	Rift Valley fever virus
SLEV	St. Louis Encephalitis Virus
PCLV	Phasi Charoen-like virus
ZIKV	Zika virus
VEEV	Venezuelan Equine Encephalitis
YFV	Yellow fever virus
WHO	World Health Organization
